# National survey: current prevalence and characteristics of home mechanical ventilation in Hungary

**DOI:** 10.1186/s12890-018-0754-x

**Published:** 2018-12-06

**Authors:** Luca Valko, Szabolcs Baglyas, Janos Gal, Andras Lorx

**Affiliations:** Department of Anesthesiology and Intensive Therapy, Semmelweis University, 1082 Üllői út 78B, Budapest, Hungary

**Keywords:** Home mechanical ventilation, Chronic respiratory failure, Home care

## Abstract

**Background:**

Home mechanical ventilation is an established treatment for chronic respiratory failure resulting in improved survival and quality of life. Technological advancement, evolving health care reimbursement systems and newly implemented national guidelines result in increased utilization worldwide. Prevalence shows great geographical variations and data on East-Central European practice has been scarce to date. The aim of the current study was to evaluate prevalence and characteristics of home mechanical ventilation in Hungary.

**Methods:**

We conducted a nationwide study using an online survey focusing on patients receiving ventilatory support at home. The survey focused on characterization of the site (affiliation, type), experience with home mechanical ventilation, number of patients treated, indication for home mechanical ventilation (disease type), description of home mechanical ventilation (invasive/noninvasive, ventilation hours, duration of ventilation) and description of the care provided (type of follow up visits, hospitalization need, reimbursement).

**Results:**

Our survey uncovered a total of 384 patients amounting to a prevalence of 3.9/100,000 in Hungary. 10.4% of patients received invasive, while 89.6% received noninvasive ventilation. The most frequent diagnosis was central hypopnea syndromes (60%), while pulmonary (20%), neuromuscular (11%) and chest wall disorders (7%) were less frequent indications. Daily ventilation need was less than 8 h in 74.2%, between 8 and 16 h in 15.4% and more than 16 h in 10.4% of patients reported. When comparing sites with a limited (< 50 patients) versus substantial (> 50 patients) case number, we found the former had significantly higher ratio of neuromuscular conditions, were more likely to ventilate invasively, with more than 16 h/day ventilation need and were more likely to provide home visits and readmit patients (*p* < 0,001).

**Conclusions:**

Our results show a reasonable current estimate and characterization of home mechanical ventilation practice in Hungary. Although a growing practice can be assumed, current prevalence is still markedly reduced compared to international data reported, the duality of current data hinting to a possible gap in diagnosis and care for more dependent patients. This points to the importance of establishing home mechanical ventilation centers, where increased experience will enable state of the art care to more dependent patients as well, increasing overall prevalence.

**Electronic supplementary material:**

The online version of this article (10.1186/s12890-018-0754-x) contains supplementary material, which is available to authorized users.

## Background

Home mechanical ventilation (HMV) is an established mode of treatment in patients with chronic respiratory failure, resulting in increased survival in several different patient groups [1–3] as well as improved quality of life and reduced hospitalization rates [4]. Use of HMV differs greatly in different parts of the world, with prevalence ranging from 2.9/100,000 in Hong Kong [5], 10.5 in Sweden [6], to 9.9–12.0 in Australia and New Zealand [7] and 12.9 in Canada [8]. The most comprehensive survey of HMV practice to date has been the Eurovent survey, although the survey mainly focused on western- and central European centers and showed a markedly reduced rate of use in the one East-Central European country reviewed (0.1 versus 6.6 overall prevalence) [9].

Since the Eurovent survey, use of this technique has been more widespread, aided by better health care reimbursement systems, improving technological supply and other advancements such as telemonitoring [10]. Many countries have created national registries, implemented national guidelines and established large HMV centers [6]. New indications have been gaining ground, with obesity hypoventilation syndrome and chronic obstructive pulmonary disease supplying an increased demand for long term mechanical ventilation [5, 7, 11].

As a result of this, current prevalence of HMV is expected to be greater than those described in the Eurovent study, even in the countries where the practice was not widespread in the last decade and organization is still lacking compared to the aforementioned nations. Poland, the only country representing the East-Central European region in the Eurovent survey reported an astonishing 116-fold increase in the number of patients treated from 2000 to 2010, with diversifying indication groups and increased prevalence of the use of noninvasive interfaces [12].

There has been no published data on HMV in Hungary, although the practice has been established since the 1990’s and has been increasingly used in recent years with the emergence of noninvasive respiratory units and increased use of noninvasive ventilation [13]. Extrapolation from the overall European prevalence of HMV from the Eurovent study would estimate about 650 patients in Hungary, not accounting for further possible increase by evolving indication guidelines, better diagnostics and improved patient recruitment.

National guidelines for HMV in the pediatric population have recently been published [14], likely improving diagnostics and care for these patients. The current Hungarian medical reimbursement system permits HMV for patients approved by the Committee of College of Health, but there are currently no assigned HMV centers.

The aim of the current study was to evaluate prevalence of home mechanical ventilation in Hungary and describe its characteristics to better aid future development of home mechanical ventilation practice in the country.

## Methods

We conducted a nationwide study in Hungary using an online survey focusing on patients receiving ventilatory support through a bilevel pressure or volume device with or without internal batteries at home under the care of a prescribing physician. Representatives of intensive care units, pulmonology centers and pediatric centers were invited to participate in the survey. Questions of the survey included characterization of the site (type of unit, yearly patient number), experience with home mechanical ventilation, number of patients treated, indication for home mechanical ventilation (disease type), description of home mechanical ventilation (invasive/noninvasive, ventilation hours, duration of ventilation) and description of the care provided (type of follow up visits, hospitalization need, reimbursement).

The study was approved by the research ethics board of Semmelweis University. Participation was voluntary and consent was implied by response to the survey. Surveys were sent out via email to all identified sites, followed by an email reminder and a telephone reminder. Survey responses were collected from March 2018 to July 2018 via an online survey program (Google Forms, Google LLC, Mountain View, United States). Sites not submitting an answer by the end of the study period were recontacted through telephone and were asked to identify the reason for non-responder status as A (“missed deadline or did not wish to submit data”) or B (“had no relevant information to share”). Returned surveys were analyzed anonymously. Data was summarized for all sites. Data are presented as median (interquartile range) for continuous and as percentages for categorical values. Relationships between sites and therapy characteristics were analyzed by Chi-squared test. Analyses were conducted using SigmaPlot 12 (Systat Software, San Jose, United States). 2018 Hungarian population data was obtained from the Hungarian Central Statistical Office [15].

## Results

Comprehensive results of the survey are provided as Additional file 1.

### Survey response rate

Overall 117 potential sites were contacted to participate in the survey. Initial response rate was 33.3% (39 sites). Telephone recontact of the sites after the initial study period showed that 91% (71) of the initially non-responder sites had no relevant information to share, while 9% (7 sites) missed the initial deadline or did not wish to participate in the survey. 47.2% (17) of sites that responded reported to actively oversee home mechanical patients, while 25% (9) provide care if needed, 13.9% (5) direct patients to other sites with more established practice. 11.1% (4) sites reported no need for HMV in any of their practice, while 11.1% (4) reported a need with inability to provide HMV. Out of the sites that responded, 72.2% (26) was aware of a HMV center, while 28.8% (10) was not. A HMV protocol was used in only 19.4% (7) sites.

### Prevalence

Overall, the 17 sites reported 384 patients receiving home mechanical ventilation, corresponding to an overall prevalence of 3.9/100.000 for home mechanical ventilation in Hungary. When looking at number of patients treated by sites, we found that 93.2% of patients were treated by four sites that had a patient number of > 50. When comparing sites with substantial case number (> 50 patients) to sites with limited case number (< 50 patients), we found that sites with a substantial case number had a significantly higher patient number (87.5(58.5;122.5) vs. 1(1;2.75); *p* = 0.002) and were more likely to be pulmonology affiliated (75% versus 0%, *p* = 0.003). Sites with a limited patient number were more likely to be intensive care unit affiliated (84.6% vs. 25%, *p* = 0.003) Table 1.

**Table 1:** Distribution of responding sites involved in HMV

	Intensive care unit affiliated	Pulmonology affiliated	Pediatric affiliated
Number of responding sites involved in HMV	12	3	2
Number of patients treated	70	306	8

### Mode of ventilation

**Fig. 1:**
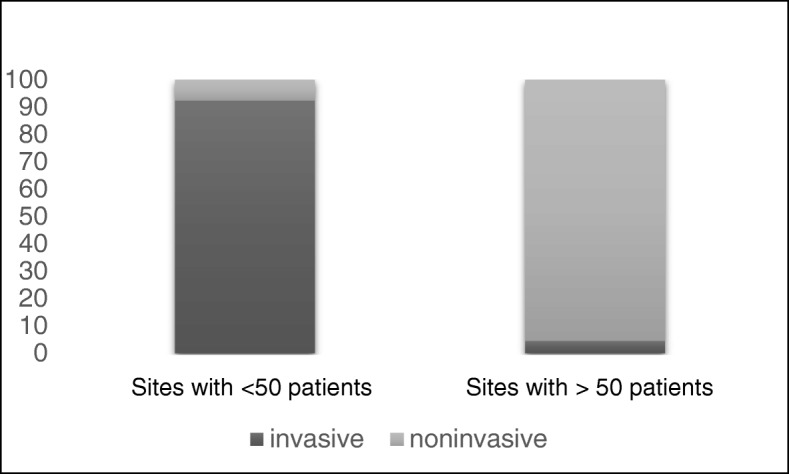
Distribution of mode of ventilation. Y axis shows percentage of patients. First column shows data from sites that care for less than 50 patients, the second column shows data from units that care for more than 50 patients. Dark shading shows patients ventilated invasively, lighter shading shows patients ventilated noninvasively.

Out of the 384 patients, 10.4% (40) received invasive, while 89.6% (344) received noninvasive ventilation. Noninvasive ventilation was used more commonly by sites with substantial case number (95.6% vs. 7.7%, *p* = 0.001), whereas invasive ventilation was the predominant mode in sites with limited case number (92.3% vs. 4.5%, *p* < 0.001) (Fig. 1).

### Indication for home mechanical ventilation

**Fig. 2:**
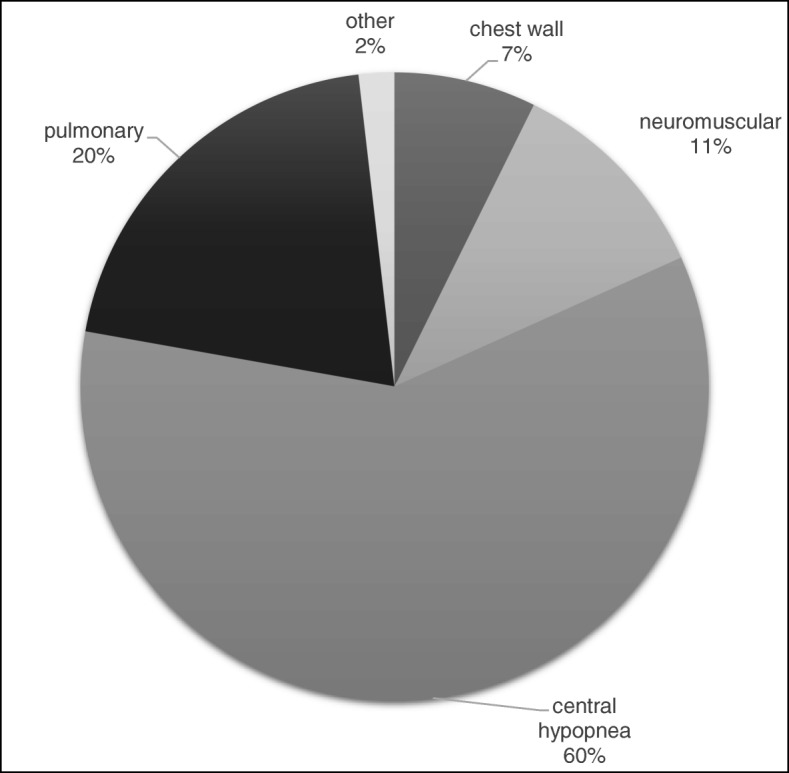
Pie chart of prevalence of indications for home mechanical ventilation

Possible indications for home mechanical ventilation need were identified as the following: central hypopnea syndromes (central alveolar hypoventilation syndrome, obesity hypoventilation syndrome); pulmonary diseases (chronic obstructive pulmonary disease, fibrosis); neuromuscular diseases (amyotrophic lateral sclerosis, systemic muscular atrophies, myasthenia, trauma related paralysis) and chest wall disorders (scoliosis, etc.) Fig. 2.

When observing the indications for sites with a substantial versus limited case number we found that most common diagnosis was central hypopnea in sites with substantial case number (62.3%) whereas neurological disease was the most frequent indication in sites with a limited case number (80%) (*p* < 0.001).

### Characteristics of home mechanical ventilation

Daily ventilation need was less than 8 h in 74.2%, between 8 and 16 h in 15.4% and more than 16 h in 10.4% of patients reported to be receiving HMV. We found that increased hours of ventilation (> 16 h/day) was more common in patients treated by a site with limited case number (80% vs. 5.6%, *p* < 0.001).

Duration of home mechanical ventilation was less than 6 months in 3.6%, 6–12 months in 9.5%, 1–5 years in 50.1%, 5–10 years in 32% and more than 10 years in 4,7% of patients reported. Distribution of duration of ventilation did not differ significantly in sites with larger versus sites with limited case number (*p* = 0,111), although there was a trend that showed a longer duration with patients treated in sites with limited experience.

### Characteristics of care provided

Follow up of patients treated with home mechanical ventilation was provided during home visits in 13.4% of cases reported, while ambulatory follow up was provided in 86.6% of cases. Home visits were more frequent at sites with limited case number compared to sites with a substantial case number (96.2% vs. 7.2%, *p* < 0.001).

Readmission rates were low overall in reported cases, with readmission needed more than twice a year in 12.6%, once a year in 4.2% and less rarely than once a year in 4.8% of reported cases. 78.4% of reported cases had no reported readmissions. When comparing sites with limited and substantial case numbers, readmission was more frequent in the former (82.9% vs. 15%, *p* < 0.001).

88.2% of sites treating home mechanical ventilation patients reported using additional devices to aid secretion elimination. Since most sites were ones treating a limited number of invasively ventilated patients, the most common reported secretion elimination method was endotracheal suction provided by 76.5% of sites, while a cough assisting device (11.8%) or both methods (11.8%) were reported to be provided by less sites (11.8 and 11.8% respectively). Notedly, cough assisting devices were only used by sites with substantial experience.

Reimbursement for HMV was either daily government reimbursement (26.4%) or initial government aid (73.5%) provided in most reported cases. Daily government reimbursement was used more frequently by sites with limited case number versus those with substantial case number (92.3% vs. 32.3%, *p* < 0.001).

## Discussion

The present study is the first comprehensive data on the use of home mechanical ventilation in Hungary. The results of our current survey show an overall prevalence of 3.9/100,000 in Hungary, with noninvasive ventilation as the most common mode of ventilation and most reported cases initiated in the last 5 years, proving the fact that HMV in Hungary has been an increasing practice in recent years. Still, the current prevalence is markedly lower than other parts of the world and even the overall prevalence of HMV in Europe identified by the Eurovent survey in 2003.

As there is no established registry for HMV and currently no assigned centers are in operation, we aimed to contact all sites possibly managing patients with failed weaning situations (intensive care units) or chronic respiratory failure patients and complex sleep related breathing disorders (pulmonology and pediatric centers). The low initial response rate of the sites contacted were thought to be indicative of the practice of home mechanical ventilation being limited to a number of sites in the country. This was verified by repeated phone contact of the non-responder sites, as 91% of nonresponding sites cited “no relevant data to share” as the reason for not completing the form.

The validity of the uncovered number of patients is further supported by reimbursement data acquired from the Hungarian National Health Insurance Fund as well as mechanical ventilator distribution data acquired from the top three distributors in the country (personal communication). As per the HNHIF, the number of patients who received active daily reimbursement for home mechanical ventilation was 97 for the month of February 2018, while an additional 102 patients were estimated to be alive who received initial government aid during the past 10 years and were not transferred to active daily reimbursement (data acquired through personal correspondence). This reimbursement data approximates a total of 199 patients receiving home mechanical ventilation in Hungary, but does not account for patients acquiring ventilators through alternative financing.

Distributor data identified a total of 244 ventilators purchased in the 10 years preceding the study period, not accounting for other potential distributors or ventilators acquired from abroad. These two alternative sources of data both provide a similar, albeit lower number of patients compared to the number uncovered by our survey, pointing to the fact that some of the patients reported in our cohort might not meet the criteria for home mechanical ventilation but rather a sleep aid device for sleep apnea, which is regarded as a different group in both reimbursement and distribution databases. Overall, this data corroborates the number of patients uncovered by our survey. More precise data collection would be possible with a national registry system.

Previous data published shows an increasing prevalence of HMV in many countries across the world [6, 11, 12, 16–18], but data is scarce on the East-Central European region. The Eurovent survey included only Poland from this region, showing a low prevalence of HMV, with patients usually treated through an invasive interface and because of a neuromuscular indication. Since then, Poland showed a remarkable improvement in patient recruitment and quality of care as well as prevalence of HMV, aided by newly established national recommendations [19].

Current practice in Hungary is still limited and can be described as two toned: intensive care units taking the burden of acutely admitted decompensated, highly ventilator dependent chronic respiratory failure patients and newly established noninvasive ventilation centers equipped with sleep labs prescribing therapy to less ventilator dependent patients but without regulated follow up. Our current results prove this duality, as the small number of sites with substantial patient numbers were significantly more likely to be pulmonology affiliated than the sites with limited patient numbers, as these were more likely to be intensive care unit affiliated.

Out of the 17 sites providing care for patients in need of home mechanical ventilation, only 4 had a patient number of more than 50 and only one unit provided care for both invasively and noninvasively ventilated patients with home visits as standard follow up care, meeting the theoretical criteria for home mechanical ventilation centers.

The relatively high ratio (89.6%) of patients receiving HMV through a noninvasive interface, is similar to recent prevalence data published from around the world [7, 8, 12], although noninvasive ventilation for home use seems to be limited to a small number of sites in Hungary.

When examining indications for HMV in Hungary, the most frequent diagnosis was central hypopnea syndromes (60%), whereas pulmonary (20%), neurological (11%) and chest wall disorder (7%) was a less frequent indication. The relative high percentage of central hypopnea cases might be due to the increased awareness of complicated sleep apnea and obesity hypoventilation syndromes and it is in par with recent data from England [20] as well as Australia and New Zealand [7].

Ventilator dependence was examined in our survey. Reported cases received ventilation mostly in less than 8 h per day, which points to the Hungarian HMV population being less ventilator dependent. Those cases with increased daily ventilation need were reported by sites with a limited case number, proving our initial theory that high ventilator dependent patients are usually initiated through an intensive care unit due to acute decompensation of chronic respiratory failure.

Quality of care of HMV patients depends on follow up visits, airway clearance methods and can be accurately described by the frequency of hospital readmissions. Our current survey on Hungarian home mechanically ventilated patients shows infrequent hospital readmission need with follow ups provided by mostly ambulatory visits. Airway clearance techniques utilized were less state of the art, mostly done by deep suctioning in patients receiving invasive mechanical ventilation, supplied by the large number of sites caring for a limited number of invasively ventilated patients. Only 23.6% of sites provided cough assisting devices for patients if needed, despite recommendations for their use in patients with reduced peak cough flows [21].

Reimbursement for home mechanical ventilation in Hungary has been reformed in 2013, with eligible patients receiving a daily funding supplied to the treatment site. Spending of funds, including choice of ventilator type, interface type and additional airway clearance devices is left to the discretion of the physician in charge of treatment, permitting a personalized treatment plan tailored to the need of the specific patient. Before 2013, government funding was available only as an initial aid in helping to obtain equipment for home mechanical ventilation often resulting in patients needing to take part in reimbursement or servicing of their equipment. Our current survey results show that despite a newer, more flexible reimbursement, the most frequently used reimbursement was still initial government aid used in 73.5% of reported cases.

When comparing sites with a limited versus larger case number, we found a clear difference. Sites caring for a limited number of patients usually managed 1 to 7 patients, were more likely to treat patients with neuromuscular indications through invasive mode, with patients requiring more than 16 h/day ventilation, home visits and more frequent readmissions. This data points to a possible gap in home mechanical ventilation provision, as patients that are more ventilator dependent but might be managed with noninvasive ventilation seem to be missing from current practice, despite recent data proving that even highly dependent, previously tracheostomized patients might be managed with continuous noninvasive ventilation [22].

The reasons for this missing group of patients can be as follows: lack of diagnosis or untimely diagnosis, misdiagnosis of patients with chronic respiratory failure and insufficient quality of care.

Lack of diagnosis or untimely diagnosis is especially prominent for patients with neuromuscular diseases, restrictive chest wall diseases and chronic obstructive pulmonary disease, when late diagnosis often results in acute hospitalization, at which point initiation of home mechanical ventilation is more difficult and results in a worse outcome [23]. Misdiagnosis of patients with chronic respiratory failure usually affects central hypoventilation syndrome patients, as these conditions are often misdiagnosed as chronic right heart failure or as simple obstructive sleep apnea, when patients only receive oxygen therapy or CPAP therapy. Our current study did not include sleep labs, nor focused on patients prescribed only long-term oxygen therapy or CPAP machines as ventilatory support, although in some of these patients HMV might be indicated with more precise work up. This points to the importance of the implementation of national guidelines on the subject. Lastly, even with timely and adequate diagnosis, insufficient care and follow up can result in worsened outcome for patients with HMV, resulting in seemingly diminished prevalence. According to our study in Hungary, so far only one established center exists that provides > 16 h/day ventilation through a noninvasive interface for the majority of its patients, state of the art secretion management devices and has a steadily growing patient number since its establishment in 2014 at Semmelweis University (data shown in supplements).

These described reasons are the most likely explanation for the still reduced prevalence of home mechanical ventilation in Hungary compared to other countries. Attempts to better identify and recruit these patients for HMV rest on establishing a system with a nationally approved adult HMV guideline, at least one center with sufficient diagnostic and follow up infrastructure and a national registry to follow care of patients already under treatment, all of which are currently evolving projects at Semmelweis University.

The main limitation of our current study is that data collection was done through a voluntary basis, possibly leading to some misidentified and some not identified cases. Overall response rate was quite low, which can be explained by the wide range of sites contacted in order to identify sites with limited patient number and experience. Another limitation of the study is that survey identification of patients and treatment characteristics is less reliable, although most published prevalence data are based on surveys conducted with similar methodology.

## Conclusion

In conclusion, our results, despite a low response rate of the survey, are the first in the country to describe current practice and based on the limited patient numbers of most responding sites, show a reasonable current estimate and characterization of home mechanical ventilation in Hungary. Although a growing practice can be assumed, current prevalence of home mechanical ventilation is still markedly reduced compared to international data reported. Our results show that currently sites with large case numbers are mainly focused on noninvasive ventilation for less ventilator dependent cases, whereas invasive interfaces are used for dependent patients with mostly neuromuscular diseases, pointing to a possible gap in diagnosis and care for more dependent patients. This points to the importance of establishing home mechanical ventilation centers, where increased experience will enable state of the art care to more dependent patients as well, increasing overall prevalence.

## Additional files

Additional file 1: Comprehensive data of responding sites. Type of site is marked as national institution (Nat), non-university hospital (NU) or university hospital (U). Affiliation is marked as pulmonary (Pulm), pediatric (Ped) or intensive care unit (ICU). Categorical questions were marked with Y (yes) or N (no). If an answer was not supplied by a site for a specific question, NA (not available) was marked. Site number 8 reported caring for home mechanical ventilation patients but currently having no patients. (DOCX 26 kb)

## Abbreviations

CPAP: Continuous positive airway pressure
HMV: Home mechanical ventilation
HNHIF: Hungarian National Health Insurance Fund
