# Supplementary material for: National survey: current prevalence and characteristics of home mechanical ventilation in Hungary
Source: BMC Pulm Med. 2018 Dec 6;18:190. doi: 10.1186/s12890-018-0754-x (PMC6282340; doi:10.1186/s12890-018-0754-x)
Supplement: Supplementary file 1 — Comprehensive data of responding sites. Type of site is marked as national institution (Nat), non-university hospital (NU) or university hospital (U). Affiliation is marked as pulmonary (Pulm), pediatric (Ped) or intensive care unit (ICU). Categorical questions were marked with Y (yes) or N (no). If an answer was not supplied by a site for a specific question, NA (not available) was marked. Site number 8 reported caring for home mechanical ventilation patients but currently having no patients. (DOCX 26 kb) [file 12890_2018_754_MOESM1_ESM.docx]

| site number | type | affiliation | number of patients | number of invasive patients | number of noninvasive patients | daily ventilation need | | | diagnosis | | | | | start of home mechanical ventilation | | | | | readmission needed | | | | reimbursement type | | | follow up type | | secretion management | |
| --- | --- | --- | --- | --- | --- | --- | --- | --- | --- | --- | --- | --- | --- | --- | --- | --- | --- | --- | --- | --- | --- | --- | --- | --- | --- | --- | --- | --- | --- |
|  |  |  |  |  |  | <8h | 8-16h | >16h | chest wall | neuromuscular | central hypopnea syndrome | pulmonary | other | <6 months | 6-12 months | 1-5 years | 5-10 years | >10 years | 1-6 months | 7-12 months | >12months | never admitted | daily government reimbursement | initial government aid | other | home visit | ambulatory visit | endotracheal suctioning | cough assist device |
| 1 | NU | Ped | 7 | 6 | 1 | 0 | 2 | 4 | 0 | 5 | 2 | 0 | 0 | 0 | 2 | 5 | 0 | 0 | 2 | 2 | 3 | 0 | 7 | 0 | 0 | Y | N | Y | N |
| 2 | NU | ICU | 1 | 1 | 0 | 0 | 0 | 1 | 0 | 1 | 0 | 0 | 0 | 0 | 0 | 1 | 0 | 0 | 1 | 0 | 0 | 0 | 1 | 0 | 0 | Y | N | Y | N |
| 3 | NU | ICU | 1 | 1 | 0 | 0 | 0 | 1 | 0 | 1 | 0 | 0 | 0 | 0 | 0 | 0 | 1 | 0 | 0 | 0 | 1 | 0 | 0 | 1 | 0 | Y | N | Y | N |
| 4 | NU | ICU | 1 | 1 | 0 | 0 | 1 | 0 | 0 | 1 | 0 | 0 | 0 | 1 | 0 | 0 | 0 | 0 | 0 | 0 | 0 | 1 | 1 | 0 | 0 | Y | Y | Y | N |
| 5 | NU | ICU | 1 | 0 | 1 | 0 | 1 | 0 | 0 | 1 | 0 | 0 | 0 | 0 | 0 | 0 | 1 | 0 | 0 | 0 | 1 | 0 | 1 | 0 | 0 | Y | N | Y | N |
| 6 | NU | Ped | 1 | 1 | 0 | 0 | 0 | 1 | 0 | 1 | 0 | 0 | 0 | 0 | 0 | 1 | 0 | 0 | 0 | 1 | 0 | 0 | 1 | 0 | 0 | Y | N | Y | N |
| 7 | NU | ICU | 2 | 2 | 0 | 0 | 0 | 2 | 0 | 2 | 0 | 0 | 0 | 0 | 0 | 1 | 1 | 0 | NA | NA | NA | NA | 2 | 0 | 0 | Y | N | Y | N |
| 8 | [NU](mailto:ngezza@hotmail.com) | ICU | 0 | 0 | 0 | 0 | 0 | 0 | 0 | 0 | 0 | 0 | 0 | 0 | 0 | 0 | 0 | 0 | 0 | 0 | 0 | 0 | 0 | 0 | 0 | NA | NA | NA | NA |
| 9 | NU | ICU | 1 | 1 | 0 | 0 | 0 | 1 | 1 | 0 | 0 | 0 | 0 | 0 | 0 | 1 | 0 | 0 | 0 | 0 | 1 | 0 | 1 | 0 | 0 | Y | N | Y | N |
| 10 | NU | ICU | 1 | 1 | 0 | 0 | 1 | 0 | 0 | 1 | 0 | 0 | 0 | 0 | 0 | 1 | 0 | 0 | 0 | 1 | 0 | 0 | 0 | 1 | 0 | Y | N | Y | N |
| 11 | Nat | ICU | 6 | 6 | 0 | 0 | 0 | 6 | 0 | 0 | 0 | 0 | 0 | 0 | 0 | 0 | 2 | 4 | NA | NA | NA | NA | 6 | 0 | 0 | Y | N | Y | N |
| 12 | NU | ICU | 1 | 1 | 0 | 0 | 0 | 1 | 0 | 1 | 0 | 0 | 0 | 0 | 0 | 0 | 1 | 0 | 1 | 0 | 0 | 0 | 1 | 0 | 0 | Y | N | Y | N |
| 13 | U | ICU | 52 | 16 | 36 | 24 | 16 | 12 | 12 | 17 | 15 | 8 | 0 | 4 | 13 | 24 | 8 | 3 | 1 | 1 | 2 | 48 | 52 | 0 | 0 | Y | Y | Y | Y |
| 14 | U | ICU | 3 | 3 | 0 | 0 | 0 | 3 | 1 | 2 | 0 | 0 | 0 | 0 | 0 | 3 | 0 | 0 | 1 | 2 | 0 | 0 | 3 | 0 | 0 | Y | N | Y | Y |
| 15 | U | Pulm | 131 | 0 | 131 | 131 | 0 | 0 | 0 | 0 | 90 | 41 | 0 | 7 | 11 | 46 | 59 | 8 | NA | NA | NA | NA | 0 | 131 | 0 | N | Y | N | Y |
| 16 | NU | Pulm | 97 | 0 | 97 | 84 | 12 | 1 | 14 | 2 | 76 | 10 | 0 | 0 | 6 | 86 | 35 | 1 | 15 | 0 | 0 | 82 | NA | NA | NA | N | Y | N | Y |
| 17 | Nat | Pulm | 78 | 0 | 78 | 45 | 26 | 7 | 0 | 7 | 45 | 19 | 7 | NA | NA | NA | NA | NA | NA | NA | NA | NA | 0 | 78 | 0 | N | Y | N | NA |
| total |  |  | 384 | 40 | 344 | 284 | 59 | 40 | 28 | 42 | 228 | 78 | 7 | 12 | 32 | 169 | 108 | 16 | 21 | 7 | 8 | 131 | 76 | 211 | 0 |  |  |  |  |

Type of site is marked as national institution (Nat), non-university hospital (NU) or university hospital (U). Affiliation is marked as pulmonary (Pulm), pediatric (Ped) or intensive care unit (ICU). Categorical questions were marked with Y (yes) or N (no). If an answer was not supplied by a site for a specific question, NA (not available) was marked. Site number 8 reported caring for home mechanical ventilation patients but currently having no patients.
